# Misophonia in a large treatment-seeking child and adolescent sample in mental health care in Germany and Austria

**DOI:** 10.1186/s12888-026-07979-1

**Published:** 2026-03-24

**Authors:** Elisa Pfeiffer, Fabienne Krech, Marc Allroggen, Ulrike M.E. Schulze, Paul L. Plener, Uschi Braun, Deniz Ercengiz, Valentin Wollenek, Nina Heinrichs, Anne Möllmann

**Affiliations:** 1 Department: Clinic for Child and Adolescent Psychiatry, Psychosomatics and Psychotherapy, Ulm University, Steinhövelstraße 1, 89075 Ulm, Germany; 2 Department of Clinical Psychology and Child and Adolescent Psychotherapy, Catholic University Eichstätt-Ingolstadt, Levelingstraße 7, 85049 Ingolstadt, Germany; 3 German Center for Mental Health (DZPG), Partner Site Ulm, Steinhoevelstr. 5, 89075 Ulm, Germany; 4 Centre for Psychiatry Calw, Department of Child and Adolescent Psychiatry and -psychotherapy, 71032 Böblingen and Weil der Stadt, Germany; 5 Department of Child and Adolescent Psychiatry, Medical University Vienna, Waehringerstr. 18-20, Vienna, 1090 Austria; 6 Department of Clinical Child and Adolescent Psychology and Psychotherapy, Bielefeld University, Universitätsstr. 25, 33615 Bielefeld, Germany; 7 Department of Psychology and Psychotherapy, Witten University, Alfred- Herrhausen-Str. 44, 58455 Witten, Germany

**Keywords:** Misophonia, Children, Adolescents, Mental health, Treatment-seeking

## Abstract

**Background:**

Misophonia is a newly conceptualized disorder. Although its onset is usually in childhood and early adolescence, the current knowledge about etiology or treatment options for children and adolescents is still limited. This is the first study investigating treatment-seeking children and adolescents in routine mental health care with regard to misophonia symptomatology and co-occurring symptoms.

**Methods:**

In this multi-center cross-sectional study, conducted in Germany and Austria, *N* = 214 participants completed measures on misophonia, anxiety, depression, posttraumatic stress disorder, obsessive-compulsive symptoms, chronic irritability, quality of life and functional impairment. The probable misophonia subsample also reported on current coping strategies and situations in which the misophonia related problems were most evident.

**Results:**

In the overall sample, 90.19% of the participants reported being bothered by at least one (misophonia-relevant) repetitive sound and 31.28% scored above the clinical cut-off for probable misophonia. In the probable misophonia subsample, 96.97% showed clinically significant co-occurring symptoms such as anxiety. The probable misophonia subsample reported significantly higher scores on all co-occurring symptom scales as well as higher functional impairment and lower quality of life, compared with the non-misophonia subsample. Most misophonia-related problems occurred in the company of other people (mostly classmates (68.18%)). The most frequent coping strategies were listening to music (57.81%) and leaving the room (25.00%).

**Discussion:**

The high rates of reported salient repetitive sounds and misophonia symptoms in routine mental health care, particularly in comparison to non–treatment-seeking populations, highlight the need for an awareness of the disorder in mental health services, and further research on feasible diagnostic and treatment approaches for misophonia and co-occurring symptoms in children and adolescents.

**Clinical trial number:**

Not applicable.

**Supplementary Information:**

The online version contains supplementary material available at 10.1186/s12888-026-07979-1.

## Highlights

First multi-center misophonia study on children and adolescents in mental health care.

(misophonia-relevant) repetitive sounds and misophonia symptoms are common in the sample.

Probable misophonia often co-occurred with internalizing symptoms.

**Supplementary Information:**

The online version contains supplementary material available at 10.1186/s12888-026-07979-1.

## Introduction

Individuals suffering from misophonia report strong negative emotional and behavioral responses to specific audiological or visual triggers, which are produced by another human. There has been an increased interest in the phenomenology, etiology and nosology of misophonia resulting in a steep increase of scientific papers in the last years [1]. A recent Delphi study, which had the overall objective of identifying and publishing a consensus definition of misophonia for the scientific community [2, 3], stated that “The Committee reached consensus to state that the symptoms of misophonia should not be better explained by auditory, psychological, and psychiatric disorders”. Apart from that, misophonia is currently not included as a distinct disorder in the latest version of the DSM-5 [6] or the ICD-11 [7].

Most current evidence regarding prevalence rates of misophonia stems from convenience student samples and/or based on online surveys [e.g. 8, 9]. These studies used various questionnaires with different thresholds, which makes general conclusions on the prevalence of the proposed disorder challenging [for an overview see 10]. Several recent representative population-based studies with adults in Ankara, Turkey [11, interview], the UK [12, interview and questionnaires], the USA [13, questionnaires] and Germany [14, 15; both questionnaires] found prevalence rates ranging between 4.6 and 18%, including categories such as “mild” or “moderate” misophonia (measured by the A-MISOS-S), indicating that the person might not suffer from clinically relevant misophonia [e.g. 13–15].

To advance the understanding of the clinical phenomenology of misophonia, studies involving psychiatric inpatient and outpatient samples are particularly informative [16–20]. For example, Siepsiak et al. [20] report that between 8.5 and 12.76% of inpatients with depression were diagnosed with misophonia. More broadly, research in treatment-seeking populations has primarily examined associations between misophonia symptoms and other psychiatric symptoms [16, 19]. These studies consistently link misophonia to internalizing symptom domains, including anxiety, depression, somatization, and obsessive–compulsive symptoms [16]. Taken together, these findings suggest that misophonia is closely embedded within the spectrum of internalizing psychopathology, highlighting the relevance of depressive and related symptoms in this context.

Although most people affected with misophonia report an age of onset in their childhood/ youth [5], surprisingly little is known regarding the clinical characteristics of misophonia in children and adolescents. This could, however, help gain a more profound understanding of the development and progression of misophonia and help to conceptualize interventions that could address the symptoms early on, to prevent later psychological impairment and reductions of quality of life. In line with studies with adults [17, 20], Guzick et al. [21] found in their sample of *N* = 102 children and youth (age 8–17 years) with misophonia in the US that most frequent misophonic trigger noises were eating, breathing or throat noises. Misophonic reactions such as annoyance/ irritation, verbal aggression and avoidance behavior were common among almost all participants. The authors found high rates of mental disorders (78% at least one), with depression and anxiety being most common. Compared to the patient group with anxiety disorders, individuals suffering from misophonia differed in regards to internalizing symptoms and symptoms of autism (both higher in the anxiety sample). Rinaldi et al. [22] found in their sample of *N* = 142 children and adolescents (*n* = 15 with probable misophonia) that children who reported misophonia above a clinical cut-off, also had significantly elevated levels of anxiety and obsessive compulsive disorder (OCD)-traits. A further analysis of the same sample showed that autistic traits were higher in children and adolescents with misophonia compared with controls without misophonia [23]. In a Polish study by Siepsiak et al. [24], participants (*N* = 90, age 7–18 years, M_age_ = 12.6; SD = 3) also reported oral human-made sounds to be their primary trigger, followed by sniffling and breathing sounds. The most common emotional responses were, similar to Guzick et al. [21], anger, irritation and disgust. Additionally, they compared children with and without misophonia and found that participants with misophonia reported significantly higher symptom rates of depression, generalized anxiety disorder, social phobia (only in self-report) and panic disorder/ agoraphobia (only in self-report). Another internalizing disorder, namely posttraumatic stress disorder (PTSD), has been found to be a highly prevalent comorbid disorder in adult patients with misophonia [4, 25], but there is a lack of research on the association of misophonia and PTSD in children and adolescents. Additionally, little is known on whether these internalizing clinical characteristics are directly associated with misophonia (treatment-seeking) children and adolescents.

As in many studies on misophonia, in both the studies by Guzick et al. [21] and Siepsiak et al. [24], the samples were recruited via online advertisements, misophonia/ anxiety-focused social media pages, online support communities and clinical/ professional networks, which could have resulted in a selection bias. The study by Lewin et al. [18] is the only study to date reporting on children and adolescents (age 8–17) who specifically sought treatment for misophonia in routine clinical care. In this study, participants were screened for enrolment in a cognitive behavioural therapy (CBT) program, and the researchers employed a combination of questionnaires and a dedicated misophonia interview. Among the 47 children and adolescents with misophonia, the most common triggers were eating, breathing, and throat sounds, consistent with findings from non-clinical samples. The predominant emotional responses to these triggers were annoyance/irritation, anger, and anxiety. Compared with Guzick et al. [21], co-occurring symptoms were reported less frequently. The most prevalent comorbid mental health disorders were generalized anxiety disorder (36.2%) and social anxiety (29.8%). Importantly, all of the studies on children have been conducted at a single site and used heterogeneous, only partially validated measures of misophonia.

### Study objectives

To date, there is no study investigating probable misophonia in a routine mental health care sample of children and adolescents and only very few studies on probable misophonia in children and adolescents in general. More evidence on probable misophonia in children in general mental health care is however crucial for a better understanding of the clinical representation of this proposed disorder in its early stages, to help practitioners in detecting misophonia cases more easily and subsequently to tailor mental health interventions to misophonia patients’ needs.

This study therefore builds on previous studies with children and adolescents with misophonia and investigates the following research aims:

1. Investigate how many children and adolescents in a treatment-seeking sample of children and adolescents in general mental health care in Germany and Austria qualify for probable misophonia.
2. Investigate whether clinical characteristics (levels of anxiety, depression, PTSD, and OCD symptoms) are associated with misophonia in a treatment-seeking sample of children and adolescents.
3. Describe misophonia and clinical characteristics of individuals with probable misophonia in regular mental health care regarding the following aspects: repetitive sounds that could be misophonia related and misophonia symptoms, age of onset, current coping strategies, the setting of misophonia-related problems, the frequency of co-occurring mental health symptoms (anxiety, depression, posttraumatic stress disorder (PTSD), OCD symptoms and chronic irritability), quality of life and functional impairment in children with probable misophonia.
4. Investigate differences between children with and without probable misophonia in regards to co-occurring mental health symptoms, quality of life and functional impairment.

## Method

The project received positive Institutional Review Board approval from the Ethics Committee of Ulm University (Number: 307/22) and the Medical University Vienna (Number: 1147/2023). The study is an anonymous questionnaire survey with children and adolescents from a general mental health care treatment seeking population in a cross-sectional study design with one measurement time point.

### Participants

Participants were eligible for inclusion if they (1) were between 10 and 21 years old, (2) sought and/ or received treatment for any mental health problem at one of the participating child and adolescent mental health care facilities, (3) were proficient in the German language to understand the questionnaires and consent forms, and (4) provided written informed study consent by themselves and their legal guardians for participants under 16 years of age. There were no exclusion criteria. The target sample size was calculated based on two approaches: Feasibility in the recruiting centers and statistical analysis. Based on previous experience with misophonia cases in the study centers in Ulm and Bielefeld, we estimated that about 40% of regular patients would fulfil study inclusion criteria for this study. Given our limited recruitment period we estimated a recruitment rate of *N* = 100 participants in Ulm and *n* = 50 participants in each center would be feasible. The recruitment period was limited by the scheduled duration of the study protocol, which included a fixed window for participant enrolment to align with treatment cycles, collaboration agreements between sites, and ethical approval timelines. This pre-specified timeframe ensured that all participants were recruited and assessed consistently across sites. The a priori power analysis with G*Power for the independent t-test requiring a power of 0.80 (estimated effect size of 0.102) and α = 0.05 (two-tailed) estimated a sample size of *N* = 102 participants. Altogether *N* = 214 children and adolescents were included in the study.

### Procedure

Study participants were recruited in outpatient and inpatient settings from four different sites in Germany and Austria: (1) Clinic for Child and Adolescent Psychiatry/Psychotherapy at Ulm University, Germany; (2) Child and Adolescent Psychotherapy Outpatient Unit (PAJU^Fam^), Bielefeld University, Germany (3) Centre for Psychiatry Calw, Department of Child and Adolescent Psychiatry and -psychotherapy, Böblingen and Weil der Stadt, Germany; (4) Department of Child and Adolescent Psychiatry at the Medical University Vienna, between November 2022 and January 2024. Children and adolescents, along with their legal guardians, were approached by trained project staff before, during or after their regular outpatient or inpatient assessment or treatment session. They were informed about the study and invited to participate. If they agreed to participate, they were provided with a written information detailing the study objectives and the voluntary nature of participation. Written consent was obtained from the children and adolescents themselves, and additionally from their legal guardians if the participants were under 16 years of age. The data was collected in paper-pencil format together with trained project staff. Trained project staff were available to address any questions or concerns during the process. The participants needed approximately 20–40 min to complete all the questionnaires.

### Measures

The **Sussex Misophonia Scale for Adolescents** (SMS-A; [22]) is utilized to assess potentially misophonia-related repetitive sounds and misophonic symptoms. The first part of the self-report questionnaire consists of 48 potential (misophonia-relevant) repetitive sounds categorized into eight overarching categories, such as “I hate people’s eating noises” which respondents can answer with “Yes” or “No”. It should be noted that misophonia is not limited to feelings of hatred or to specific sound categories. If a category is answered affirmatively, respondents are then presented with a corresponding list of specific sounds, such as “crunchy snacks” or “swallowing,” which they should indicate if applicable.

In the second part, misophonic symptoms are assessed using 39 items rated on a five-point Likert scale ranging from *never* (0) to *always* (4). These items include statements regarding difficulties with certain sounds. A total sum score of 49 or higher indicates the presence of misophonia, as suggested by Rinaldi et al. [22]. If the participant did not report any repetitive sound, part 2 was not completed. Additionally, to explore the reported sounds, the items of SMS-A Part 1 are summed up, and absolute and relative frequencies of each sound group are calculated. The questionnaire was translated for this project in accordance with current translation guidelines by the WHO [26] and with the consent of the authors, led by Dr. Rinaldi. The German version of the measure has not been validated yet. The self-report of misophonic symptoms demonstrated excellent internal consistency (Cronbach’s α = 0.97) in this study.

Misophonia symptoms are further explored via self-generated questions. Participants are asked about the age of onset of their misophonic symptoms. They are then prompted to describe possible coping strategies in an open question. Participants also specify the contexts in which their problems associated with the repetitive sounds are most severe, such as when they were alone or with others, including details about the presence of which people who exacerbated their symptoms, allowing for multiple answers. These questions are only required if the second part of the SMS-A was completed.

The **Mood and Feelings Questionnaire: Short Version** (SMFQ; [27]) is used to assess depressive symptoms using 13 items. The items, such as “I felt bad or unhappy”, record the depressive symptoms in the last two weeks on a three-point Likert scale from *not true* (0) to *true* (2) [27]. According to Angold et al. [27], a value of 8 or higher indicates the presence of clinically relevant depression. In the present study, the SMFQ demonstrated excellent internal consistency (α = 0.934).

The short version of the **Child and Adolescent Trauma Screen 2** (CATS-2; [28]) is used to assess symptoms of PTSD. In the first part of the questionnaire, traumatic experiences are assessed, followed by PTSD symptoms using 6 items, such as “Images of the event come into my head. I have the feeling that it will happen again.”, referring to the past four weeks [28]. The symptom items are answered on a four-point Likert scale from *never* (0) to *almost always* (4). If no traumatic experiences were reported in the first part of the questionnaire, the other parts did not need to be completed. According to Sachser et al. [28] a total score of 7 or higher indicates increased PTSD symptoms and a score of 9 or higher indicates clinically relevant PTSD. In this study the measure had good internal consistency (α = 0.854).

The short version of the **Screen for Child Anxiety related Disorders** (SCARED; [29]) is used to assess anxiety symptoms on a three-point Likert scale from *not true or rarely true* (0) to *true or often true* (2). Items include general statements about anxiety, such as “I get anxious for no reason at all.” [29]. According to Birmaher et al. [29], a total score of 3 or higher indicates the presence of clinically relevant anxiety. The internal consistency in this study was α = 0.664.

The **Obsessive-Compulsive Inventory for Children and Adolescents - Self-Report** (ZWIK-S; [30]) is used to measure OCD symptoms (obsessive thoughts and compulsive actions; 36 items) on a five-point Likert scale from *not at all* (0) to *very strongly* (4), with items like “I wash my hands more often and for longer than necessary.” [30]. A total score of 36 or higher indicates clinically relevant OCD [30]. In this study the ZWIK-S demonstrated excellent internal consistency (α = 0.956).

The **Affective Reactivity Index** (ARI; [31]) measures chronic irritability on a three-point Likert scale from *not true* (0) to *certainly true* (2). The first six items capture specific feelings/behaviors of irritability (e.g. “I often get angry. “) and the last item captures the impairment due to irritability (“Overall, I get a lot of problems because of my irritability.”). Higher scores indicate greater irritability [31]. In this study, the ARI demonstrated good internal consistency (α = .887).

The **KIDSCREEN-10** questionnaire [32, 33] is used to measure quality of life (10 items) on a five-point Likert scale from *not at all* (1) to *very much* (5), such as “Did you feel fit and well?” [33]. The raw scores are transformed into T-scores, with higher scores indicating a higher quality of life [32]. In this study, the KIDSCREEN-10 demonstrated good internal consistency (α = 0.802).

The **Work and Social Adjustment Scale** (WSAS-Y; [34], German translation: [35]) is used to record functional impairment (5 items) in the areas of school/work, everyday activities, social activities, leisure and family/relationships on a nine-point Likert scale from *not at all* (0) to *very severely* (8), such as “Think about whether you can meet friends when you feel like it, e.g. to spend the night with them, to go shopping, out to eat or to the movies, or to do something after school. How does your problem affect these areas?”. Higher scores indicate a higher functional impairment. In this study, the WSAS-Y demonstrated good internal consistency (α = 0.849).

### Statistical analysis

Missing values were imputed at the item level, allowing up to 20% missing values per questionnaire, using the subscale mean. One participant was excluded from the study sample as he/ she had more than 20% missing data in the questionnaires. Additionally, *n* = 3 cases had to be excluded from the sub-sample analysis because they did not report any (misophonia-relevant) repetitive sounds on the SMS-A checklist and did not complete the items on misophonic symptoms.

Descriptive statistics were computed to describe the sociodemographic characteristics of the participants and questionnaires (research question 1 and 3). In addition, chi-squared tests were calculated to investigate group differences. Coping strategies were clustered into 10 categories (listen to music, headphones, earplugs, leaving room / situation, distraction, endure/wait until it is over, avoidance/withdrawal, scream, other, nothing).

For answering the second research question, we calculated a Pearson moment correlation after checking all prerequisites. We then conducted a multiple regression analysis with depression, anxiety, PTSD and OCD as continuous independent variables and misophonia symptom scores as the dependent variable. All statistical prerequisites were met, but *n* = 2 were excluded due to outliers in the SMS-A (+ 3SD). For answering our fourth research question, group differences between children with and without probable misophonia were calculated using independent t-tests. If the assumption of homogeneity of variances was not met, the Welch test was conducted. A test of normal distribution was omitted as the sample size was larger than 30 [see recommendation of e.g. 36, 37]. To mitigate Type I errors, the False Discovery Rate was employed as a correction procedure. The significance level was set at *p* <.05. Effect sizes (Cohen’s *d*) were calculated for comparisons. Data analyses were conducted using IBM SPSS Statistics for Windows, Version 28.0.

## Results

The study sample comprised *N* = 214 children and adolescents. The demographic information is presented in Table 1.

**Table 1:** Age and Gender distribution in the total sample and sub-samples of children and adolescents with and without probable misophonia

	Total sample *N* = 214	With Probable Misophonia *n* = 66	Without Probable Misophonia *n* = 145
Age	M = 14.73 (SD = 2.16, range = 10–21)	M = 14.64 (SD = 2.02, range = 10–20)	M = 14.76 (SD = 2.17, range = 10–21)
Gender n (%)			
female male non-binary/ genderqueer no gender not specified other	121 (56.54) 70 (32.71) 9 (4.21) 1 (0.47) 7 (3.27) 6 (2.80)	42 (63.63) 13 (19.69) 3 (4.55) 1 (1.51) 3 (4.55) 4 (6.06)	78 (53.79) 55 (37.93) 6 (4.14) 0 (0.00) 4 (2.76) 2 (1.37)

### Characteristics of repetitive sounds and misophonia symptom severity in the overall study sample

**Fig. 1:**
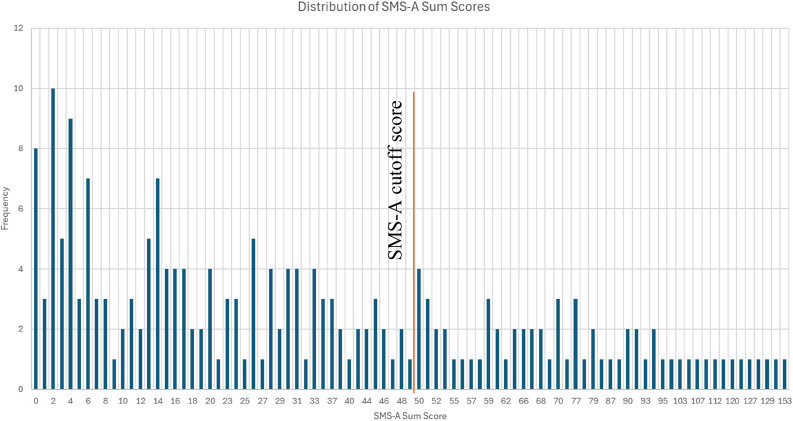
Frequency distribution of the sum scores of the SMS-A (total sample)

Regarding (misophonia-relevant) repetitive sounds, *n* = 193 (90.19%) reported at least one sound (see Table 2). The mean misophonic symptom score was 36.95 (*SD* = 33.84, range = 0-153). A sub-sample of *n* = 66 participants (31.28%) met the SMS-A cutoff criterion of > 49 indicating probable misophonia. The frequency distribution of the sum scores of the SMS-A is also shown in Fig. 1. For a distribution of misophonia prevalence as a function of age please see Fig. 1 in the supplement.

**Table 2:** Categories of repetitive sounds in the treatment-seeking study sample and sub-samples

	Total sample *N* = 214	With probable Misophonia *n* = 66	Without probable Misophonia *n* = 145	Group differences		
				χ²(1)	*p*	φ
Sound of people eating	159 (74.30)	62 (93.94)	97 (66.90)	17.86	*< 0.001*	0.29
Sounds people make through their mouth and nose	153 (71.50)	64 (96.97)	89 (61.38)	28.82	*< 0.001*	0.37
Background sounds	145 (67.76)	58 (87.88)	87 (60.00)	16.40	*< 0.001*	0.28
Sound of repetitive tapping	118 (55.14)	57 (86.36)	61 (42.07)	36.10	*< 0.001*	0.41
Voice sounds	109 (50.93)	46 (69.70)	63 (43.45)	13.42	*< 0.001*	0.25
Throat sounds	85 (39.72)	45 (68.18)	40 (27.59)	31.07	*< 0.001*	0.38
Repetitive visual movements	82 (38.32)	44 (66.67)	38 (26.21)	31.25	*< 0.001*	0.39
Sound of rustling	57 (26.64)	32 (48.48)	25 (17.24)	22.93	*< 0.001*	0.33

*Notes. n* (% in brackets). Sub-samples according to the SMS-A cutoff criterion of > 49

### Association between clinical characteristics and misophonia

Pearson moment correlations were first calculated to examine the association between clinical characteristics and misophonia symptoms as a function of a continuous variable. All clinical characteristics significantly correlated with misophonia symptoms (see online supplement Table S1). Depression and OCD symptoms significantly predicted misophonia symptoms (see Table 3). Overall, the model had a high variance explanation and was statistically significant (R² = 0.37, *F*(4,171) = 25.06, *p* < .001.)

**Table 3:** Summary of multiple regression analysis predicting misophonia symptoms from clinical characteristics

Predictors	B	SE	β	t	*p*	95% CI for B
(Constant)	3.05	4.32		0.71	0.481	[-5.47, 11.56]
*Depression*	0.77	0.37	0.17	2.10	0.037*	[0.05, 1.49]
*Anxiety*	1.22	1.12	0.09	1.08	0.281	[-1.00, 3.43]
*PTSD*	1.02	0.57	0.15	1.78	0.076	[-0.11, 2.15]
*OCD*	0.37	0.09	0.33	4.13	< 0.001***	[0.19, 0.55]

Notes.* *p* < .05. ** *p* < .01. *** *p* <.001

### Description of the participants meeting the SMS-A cutoff criterion (probable misophonia subsample)

**Fig. 2:**
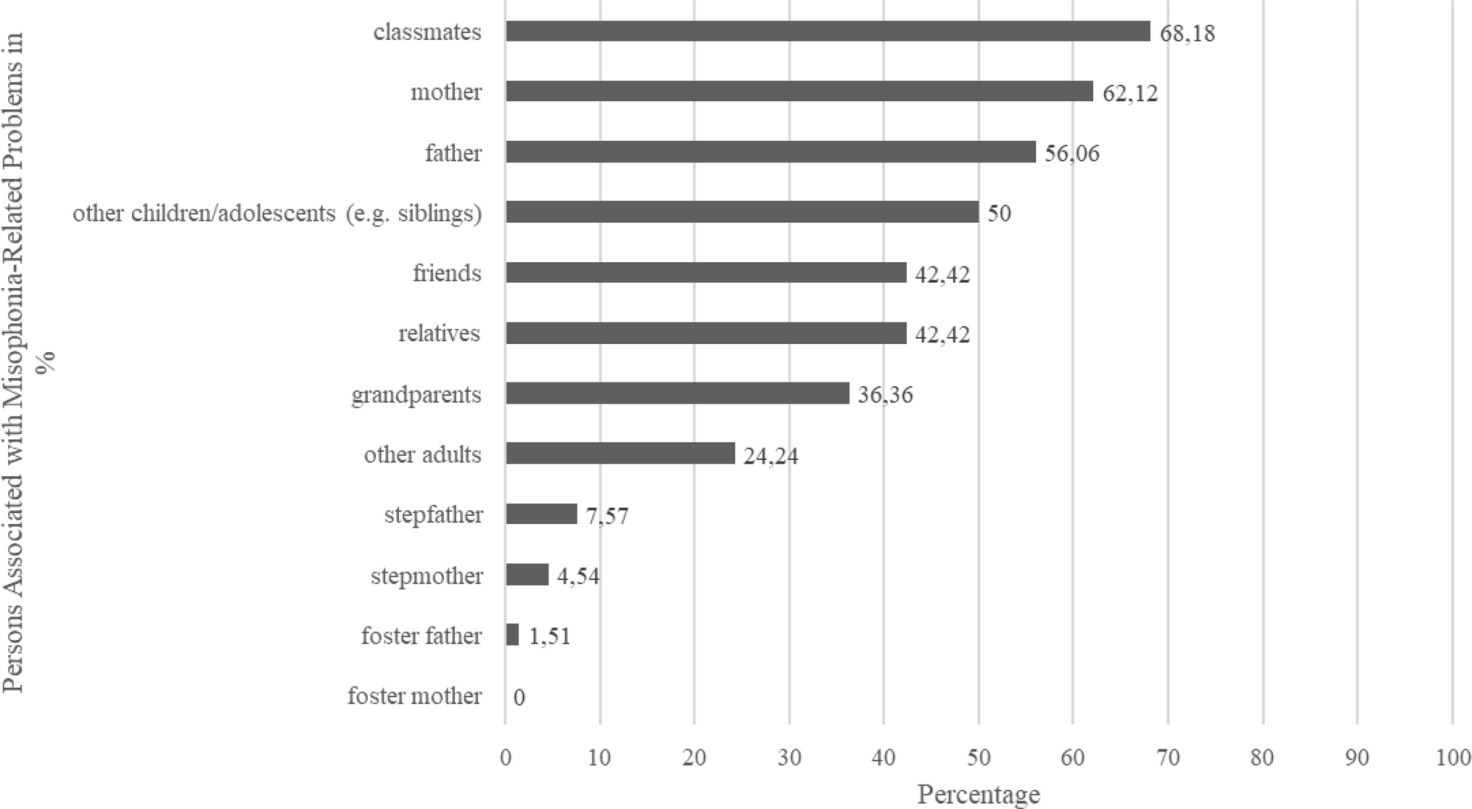
Distribution of persons associated with misophonia-related problems in % for subsample of children and adolescents with probable misophonia

**Fig. 3:**
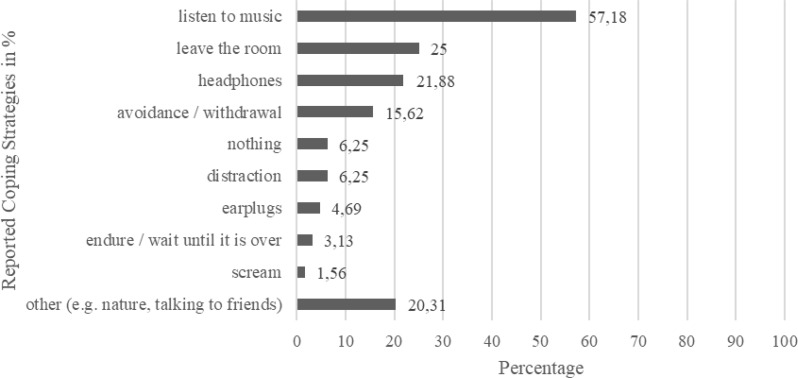
Distribution of reported coping strategies in % for the subsample of children and adolescents with probable misophonia (*n* = 66). *Note.* Open question format. Participants could report several coping strategies

The 66 children and adolescents with probable misophonia reported *M* = 6.18 (*SD* = 1.56, range = 2–8) different misophonia-relevant repetitive sounds. Sounds people make through their mouth and nose were the most common (96.97%), followed by the sound of people eating (93.94%) and background sounds (87.88%; see Table 2). The probable misophonia group reported on average a symptom load of *M* = 79.07 (*SD* = 25.47, range = 49–153). The mean age of onset was 9.39 years old (*SD* = 3.02, range = 3–16 years). Regarding the setting of problems associated with the repetitive sounds, *n* = 54 (83.08%) reported experiencing the most severe problems with sounds when they were with other people, *n* = 4 (6.15%) reported experiencing them when they were alone, and *n* = 7 (10.77%) reported experiencing them in both situations (*n* = 1 participant did not answer this item). The most frequently reported persons involved were parents and classmates (see Fig. 2). Please note that participants could indicate several persons at the same time. Most participants with probable misophonia reported that they use music (57.81%) or leaving the room (25.00%) as a coping strategy. Please see Fig. 3 for more reported coping strategies.

Most participants in the probable misophonia group (96.97%) reported clinically relevant co-occurring other symptoms. On average, the probable misophonia group scored in *M* = 2.98 (*SD* = 1.15, range = 0–4) other symptom areas above the respective clinical cut-offs, most frequently anxiety (84.85%), followed by depression (78.79%), PTSD (68.18%) and OCD (66.67%) (see Online Supplement Table B). The probable misophonia group reported a mean of *M* = 5.16 (*SD* = 3.33) in irritability and a mean of *M* = 19.48 (*SD* = 7.89) in functional impairment.

### Differences between children with and without probable misophonia

As expected based on the group definition, the probable misophonia group reported a higher number of repetitive sounds ((*M*_*non−misophonia group*_ = 3.45; *SD* = 2.14, range = 0–8; *t*(168.729) = 10.445, *p* < .001; *d* = 1.38) and misophonic symptoms (Table 4), compared with the non-misophonia group. These variables were used to characterize the groups descriptively, whereas subsequent analyses focused on differences in co-occurring symptoms, functional impairment, and quality of life (see Table S2).

Regarding co-occurring symptoms, the probable misophonia group reported a higher number of clinically elevated symptom areas (*M*_*non−misophonia group*_ = 1.79 (*SD* = 1.34, range = 0–4); *t*(144.136) = 6.598, *p* < .001, d = 0.93) and higher symptom severity on all symptom severity measures (depression, PTSD, anxiety, OCD), as well as lower quality of life and higher functional impairment (please see Table 4). The probable misophonia group reported significantly lower quality of life scores and higher functional impairment compared to the non-misophonia group (see Table 4). For an overview of the frequency of symptoms of depression, PTSD, anxiety, and OCD in the Subsamples with/ without Probable Misophonia, please see Table B in the appendix.

**Table 4:** Means, standard deviations and ranges of children and adolescents with and without probable misophonia as well as the total sample for age, misophonic symptoms, quality of life, symptoms of depression, PTSD, anxiety, and OCD

	Total Sample (*N* = 211)	With probable Misophonia (*n* = 66)	Without probable Misophonia (*n* = 145)	Group differences			
	M (SD); range	M (SD); range	M (SD); range	t	df	*p*	d
Misophonic Symptoms (SMS-A)	36.95 (33.84); 0–153	79.07 (25.47); 49–153	17.77 (13.91); 0–48	*18.34*	*83.162*	*< 0.001*	*3.35*
Depressive Symptoms (SMFQ)	11.38 (7.58); 0–26	15.29 (7.44); 3–26	9.71 (6.96); 0–24	*5.28*	*209*	*< 0.001*	*-0.79*
PTSD Symptoms (CATS-2)	6.94 (4.91); 0–18	9.28 (4.83); 0–18	5.78 (4.51); 0–16	*4.86*	*180*	*< 0.001*	*-0.76*
Anxiety Symptoms (SCARED)	3.92 (2.47); 0–10	5.00 (2.27); 0–10	3.46 (2.40); 0–10	*4.33*	*206*	*< 0.001*	*-0.65*
Irritability (ARI)	3.90 (3.38); 0–12	5.16 (3.33); 0–12	3.36 (3.26); 0–12	*3.65*	*206*	*< 0.001*	*-0.55*
OCD-Symptoms (ZWIK-S)	37.19 (28.87); 1-144	54.05 (35.23); 8-144	29.84 (21.61); 1–96	*5.12*	*86.774*	*< 0.001*	*-0.91*
Quality of Life (KIDSCREEN-10)	39.05 (5.21); 26.64–66.86	36.33 (6.62); 18.50–51.36	41.16(8.77); 18.50–83.81	*4.36*	*158.072*	*< 0.001*	*0.60*
Functional Impairment (WSAS-Y)	14.45 (9.67); 0–39	19.48 (7.89); 4–39	12.39 (9.55); 0–38	*5.60*	*144.598*	*< 0.001*	*-0.78*

## Discussion

This is the first multi-center study on the phenomenology of misophonia in children and adolescents in routine mental health care, assessing children in both inpatient and outpatient settings. One of the most important findings of this study is the high prevalence of (potentially misophonia-relevant) repetitive sounds in the general treatment-seeking sample. Almost all children (90.19%) reported to hate at least one repetitive sound. Moreover, with exception of the rustling sound (26.64%), all listed sounds were reported by more than half of the participants. The most often reported repetitive sounds in this current study by the general and the probable misophonia sub-sample (eating/ mouth/ nose sounds) are in line with most of the previous misophonia studies [5, 12]. This finding supports the recently postulated idea that sensitivity to these specific repetitive sounds may reflect misophonia as a dimensional construct [10] and might be highly prevalent not only in the general population [13, 15] but also in children and adolescents with mental health seeking behavior in particular [5, 17]. Importantly, the high frequency of disliked sounds in the overall sample underscores the need to distinguish between common sound aversion and clinically significant misophonia. According to the neurophysiological model proposed by Jastreboff & Jastreboff [38], which is based mostly on clinical observations, misophonia reflects abnormally strong autonomic and limbic reactions resulting from enhanced functional connectivity between auditory, limbic, and autonomic systems for particular patterns of sound. The authors argue that there is nothing inherently specific about the sounds themselves; rather, the “specificity” refers to the relationship between a given sound and a given individual. In principle, any sound can become a misophonic trigger if it acquires heightened salience within this neurobiological network. This perspective aligns with our findings that many repetitive sounds are broadly disliked, whereas only a smaller subgroup exhibits probable misophonia characterized by clinically relevant symptomatology.

Not only were the disliked repetitive sounds highly prevalent in our sample, but 31.28% reported misophonic symptoms above the clinical cut-off. This finding is noteworthy as the clinical cut-off at 49 was chosen conservatively by the developers of the measure, with a threshold at the 90th percentile of total SMS-Adolescent scores derived from a sample outside of the mental health care setting Rinaldi et al. [22]. This implicates strong differences in the presence of misophonic symptoms between clinical and non-clinical samples. The frequency distribution of the SMS-A sum scores (Fig. 1) in the full sample further showed that scores were not normally distributed but instead skewed towards lower scores being more frequent and high scores being rather evenly distributed. Similar findings in which many (adult) participants report (misophonia-relevant) repetitive sounds and low/ mild symptoms, but comparably little participants report moderate to severe or extreme symptoms were found in a population-based study in Germany [15].

We found strong and statistically significant correlations between misophonia and other internalizing symptoms (PTSD, depression, anxiety, OCD), which is in line with the current evidence-base on comorbidity of misophonia [e.g. 16]. Depression and OCD symptoms also predicted higher misophonia symptom burden. Yet, a significant proportion of variance remained unexplained, supporting the idea that misophonia may represent a distinct construct beyond established diagnoses [3, 5, 11, 17, 39]). Depression symptoms remained predictive even after controlling for anxiety, PTSD, and OCD, in line with previous findings [19, 40]. However, given the cross-sectional design of the present study, no conclusions can be drawn regarding the direction of this relationship. It is equally plausible that misophonia-related distress and functional impairment contribute to depressive symptoms, or that both share common underlying vulnerability factors. Interestingly, OCD symptoms were more strongly associated with misophonia severity in children and adolescents than in adults [11, 19, 40]. This age-related difference may reflect developmental variations in regulatory capacities or coping strategies in response to distressing sensory stimuli. Behaviors such as avoidance or repetitive self-regulation in the context of misophonic sounds/ triggers should not be equated with compulsions in the classical sense of obsessive–compulsive disorder (OCD). Rather, they may represent attempts to manage heightened sensory sensitivity or emotional reactivity. Recent research on sensory processing in paediatric OCD and anxiety [41, 42] suggests that sensory over-responsivity and difficulties in sensory modulation may contribute to symptom expression. Within this framework, the observed association between misophonia and obsessive–compulsive symptoms may reflect partially overlapping mechanisms related to sensory processing, rather than diagnostic misclassification.

The probable misophonia subsample reported extremely high rates of (misophonia-relevant) repetitive sounds and symptoms which shows their high symptom burden. The probable misophonia sample reported high rates of co-occurring other symptoms with 97% of them reporting symptoms above the clinical cut-off of at least one other condition. This finding is, however, not surprising as all participants were generally treatment-seeking (not specifically for misophonia). The high rates of internalizing symptoms support the notion that misophonia may be seen as an internalizing disorder or often co-occur with these disorders [24]. In fact, a recent study by Guetta et al. [43] investigated the association between misophonia, stress, lifetime traumatic events and posttraumatic stress symptoms in adults with misophonia. Based on their findings, they suggest a transdiagnostic process related to stress to be an important etiological factor of misophonia as perceived stress and hyperarousal predicted misophonia (not trauma or PTSD in general). Perceived stress and symptoms of hyperarousal play a role in all internalizing disorders which could thus explain the high comorbidity of misophonia with these symptoms.

On closer inspection, the rates of clinical depression, anxiety, PTSD and OCD symptoms are even higher in our sample compared to Guzick et al. [21] and Lewin et al. [18], which could be explained by methodological differences in the studies such as the measures employed. Rates of PTSD were also significantly higher compared with recent adult studies [4, 25]. Alternatively, the high prevalence rates could be explained by the fact that participants in this study were all treatment seeking for a mental health condition and part of the sample was currently treated in an inpatient setting. The probable misophonia group reported higher symptom severity compared with the non-misophonia group across all other conditions which has been seen in several previous misophonia studies [e.g. 22]. The strongest difference could be found in OCD symptoms, which is contrary to the study by Siepsiak et al. [24] who found no difference for this co-occurring symptom area in their sample when assessed in child self-report measure. They did find a difference when assessed in an interview according to ICD-10 criteria though. This difference could potentially be explained by the different questionnaires used as we employed a longer and more comprehensive measure in the current study. The significantly lower functioning and Quality of Life in the children with probable misophonia has also been reported by Rinaldi et al. [22] and seems a logical consequence of their general high mental health symptom burden.

The age of onset of 9.4 years in our sample was very similar to the study by Lewin et al. [18] (9.2 years) and supports Siepsiak et al. [24] recent finding that misophonia may begin even earlier than previously reported [4, 13]. Similarly, Rouw and Erfanian [5] revealed that 45% of participants in their large adult online-survey sample reported misophonia symptom onset in childhood (5–12 years old) and 15% of the participants reported that they had symptoms “As long as I can remember” (answers referring to 2, 3, or 4 years of age). Hence, early detection of misophonia in childhood and adolescents may be especially crucial to prevent or reduce adverse long-term effects of misophonia.

Most of the children with probable misophonia report experiencing sounds/ triggers primarily when being with another person (83.08%) which is not surprising as most of the sounds are human-generated by definition. Interestingly, a small subgroup (6.15%) report that they experience disliking of these sounds (also) when alone suggesting that symptom activation may not be limited to direct interpersonal contexts and may reflect constant exposure to misophonic triggers in everyday environments. In their social-cognitive model of misophonia, Berger et al. [44] propose that misophonia should be understood within the broader framework of social perception and social cognition. According to this model, neurobiological and physiological responses to triggering (misophonic) sounds are shaped not only by acoustic features but also by contextual and interpersonal factors, such as who produces the sound and how it is interpreted. In light of our findings, future research should systematically examine the role of the social context in misophonia symptom activation, for example by investigating whether emotional and physiological responses differ depending on the relationship to the sound source (e.g., family member classmate) or whether symptoms persist in socially neutral contexts. Such studies may help clarify the extent to which misophonic reactions are driven by sensory processing alone versus socially mediated cognitive-affective mechanisms.

Similar to the study by Siepsiak et al. [24] we found that the problems related to the misophonic sounds were perceived as particularly strong when performed by close family members (especially parents), but this study crucially extends current evidence as we found that many children were also triggered by their classmates (68.18%), other children/ adolescents (including siblings, 50.00%) and friends (42.42%). Other family members (e.g. foster – or step-parents) and adults reside on lower percentages, most likely because only few participants might have a foster- or step-parent. The high prevalence rates of peers highlight the reality of children and adolescents nowadays in which they often spend more time outside of their home with peers, potentially resulting in these peers being an especially frequent trigger person. This finding needs to be investigated further, to be able to form any conclusions on the impact of peer relationships on misophonia in children and adolescents. Especially the impact of (cyber) bullying and abilities to social and cognitive judgements [45] could be a target of future investigations.

To our knowledge, this is the first study investigating current coping strategies in children and adolescents with misophonia symptoms. These skills are employed by the children without misophonia-specific evidence-based treatment as such treatment is not available in Germany or Austria yet. They reported that withdrawing themselves from the situation (listen to music, headphones or leaving the room altogether) was most often their strategy of choice. On the one hand, these findings demonstrate how resourceful these children are in developing their own ways to navigate daily challenges in the absence of misophonia-specific treatment. On the other hand, avoiding the triggers may result in an exclusion from various situations with others which could potentially result in less opportunities for a healthy socio-emotional development.

### Limitations

This study presents several limitations that should be considered when interpreting the results: (1) This study does not constitute a population-based prevalence study. The sample consisted exclusively of treatment-seeking children and adolescents in mental health care services, and the results therefore reflect the proportion of probable misophonia within this specific clinical context rather than prevalence in the general population or in routine care settings more broadly. Additionally, recruitment bias may have influenced the observed proportion. Two out of the four study sites offered specific outpatient consultations for misophonia (mostly psychoeducation), which may have led some families to seek assessment at these centers due to their known expertise in this area. As data were anonymized to the greatest extent possible, systematic identification of such referral pathways was not feasible. An unsystematic survey among assessors suggested that this may have applied to approximately 10–15 participants. Given the total sample size of 214 children and adolescents, this represents a relatively small proportion of the overall cohort; nevertheless, selective referral cannot be ruled out and may have led to a modest overestimation of probable misophonia within this sample. (2) Contrary to previous studies on children with misophonia [18, 21, 24], this study only used self-report data due to feasibility reasons. Not having incorporated clinical interviews limits the objectivity of the data and prevents any firm conclusions regarding mental health diagnoses. (3) The SMS-A measure is currently only validated in the original English version, not in this German version. (4) The list of (misophonia-relevant) repetitive sound*s* might not fully represent all potential misophonic triggers (e.g. sounds through wall/ceiling/floor are missing. 5) The coping strategies were assessed in an open question in which participants could write several strategies without given examples. 6) The answers on the “trigger persons” item should be interpreted with caution as the item allowed for multiple answers and assessed with whom the symptoms were the worst instead of all potential trigger persons. Additionally, broader categories such as “other children/ adolescents” did not systematically assess specific categories of other children (e.g. siblings, cousins etc.) and “classmates” could include several persons, whereas categories such as “mother” only include one person. 7) The Cronbach’s alpha for the SCARED questionnaire in the present sample was below the generally accepted threshold (< 0.7), indicating lower internal consistency. This limitation may have reduced the reliability of anxiety symptom measurements and could have attenuated observed associations. Therefore, results involving SCARED scores should be interpreted with caution. 8) Due to the anonymized data and the study being independent of their regular mental health care, their current clinical diagnosis or symptom severity outside of the study data is not reported. The definition of this sample being a treatment-seeking (clinical) population, rather than a community sample, based on the fact that all participants were currently receiving mental health care services. 9) In the overall project we also collected data on other forms of noise sensitivity and used the Highly Sensitive Child Scale (HSC; [46]) to assess environmental sensitivity of children. As the measure was included later during the ongoing study and only two study centers could still add the measure to their data collection given the late changes, this data is not reported in this manuscript. 10) The response rate for participation and the exact wording used in solicitation of participants were not systematically recorded. Hence a potential self-selection bias with greater percentage of those with misophonia symptoms participating in this study cannot be ruled out.

### Clinical implications and future research

Although almost all participants report to hate at least one (misophonia-relevant) repetitive sound, the majority does not report misophonic symptoms above the clinical cut-off. Hence, experiencing discomfort in response to these repetitive sounds does not necessarily indicate a diagnosis of misophonia. The findings on the early onset of probable misophonia in our sample and the observed high symptom burden highlight the need of early detection of misophonia in the mental health care system as this may mitigate adverse long-term effects of misophonia on the child’s mental health and psycho-social functioning. Misophonia assessment tools need to be further validated, translated, and disseminated. Due to the early onset of the condition, these measures need to be feasible for children of a young age as well. In light of the high rate of co-occurring symptoms in children with probable misophonia, screening for other mental health disorders should be part of a misophonia diagnostic process. From a clinical as well as research perspective, it appears important to improve the understanding of the interplay between the many different areas of co-occurring symptoms, for example with a network analysis approach. Considering our sample of youth, who were seeking treatment for a variety of reasons and revealing different co-occurring symptoms, we do not know which symptoms are more likely to occur as a result of or as a preceding symptom of probable misophonia and vice versa. Future studies might thus investigate (a) whether higher misophonic symptom severity is similarly present in treatment-seeking youth with versus without misophonia as their main concern, (b) whether (or under which circumstances) misophonia symptoms decrease during treatment of other mental health conditions, and (c) whether (or how) other symptoms are affected by misophonia specific treatment.

## Conclusion

The high prevalence of repetitive sounds and misophonia symptoms in this sample highlights the need for sensitivity of this disorder among health care practitioners to not only identify these children in routine care, but to then offer them a mental health care intervention that fits their needs. Next to an extensive evaluation of potential treatment approaches specifically for children with misophonia, the dissemination of these is of utmost importance.

## Supplementary Information

Below is the link to the electronic supplementary material.

Supplementary Material 1

## Data Availability

The datasets used and/or analyzed during the current study are available from the corresponding author on reasonable request.

## Abbreviations

ARI: Affective Reactivity Index
CATS-2: Child and Adolescent Trauma Screen 2
OCD: Obsessive compulsive disorder
PTSD: Posttraumatic stress disorder
SCARED: Screen for Child Anxiety related Disorders
SMFQ: Mood and Feelings Questionnaire: Short Version
SMS-A: Sussex Misophonia Scale for Adolescents
WASAS-Y: Work and Social Adjustment Scale
ZWIK-S: Obsessive-Compulsive Inventory for Children and Adolescents - Self-Report
