# Supplementary material for: Misophonia in a large treatment-seeking child and adolescent sample in mental health care in Germany and Austria
Source: BMC Psychiatry. 2026 Mar 24;26:292. doi: 10.1186/s12888-026-07979-1 (PMC13064043; doi:10.1186/s12888-026-07979-1)
Supplement: Supplementary file 1 — Supplementary Material 1 [file 12888_2026_7979_MOESM1_ESM.docx]

Online Supplement

**Table S1**

*Pearson-Moment-Correlations Between Misophonia Symptoms and Clinical Characteristics.*

| Variable | 1 | 2 | 3 | 4 | 5 |
| --- | --- | --- | --- | --- | --- |
| 1. Misophonia | 1 |  |  |  |  |
| 2. Depression | .46*** | 1 |  |  |  |
| 3. Anxiety | .44*** | .61*** | 1 |  |  |
| 4. PTSD | .49*** | .56*** | .52*** | 1 |  |
| 5. OCD | .54*** | .44*** | .51*** | .60*** | 1 |

*Note.* *** indicates *p* < 0.001

**Table S2**

Frequency of Symptoms of Depression, PTSD, Anxiety, and OCD in the Subsamples with/ without Probable Misophonia.

|  | With Probable Misophonia  *n* = 66 | Without Probable Misophonia  *n* = 145 |
| --- | --- | --- |
| Anxiety (FAS-K) | 56 (84.85) | 88 (60.69) |
| Depression (SMFQ) | 52 (78.79) | 79 (54.48) |
| PTSD (CATS-2) | 45 (68.18) | 49 (33.79) |
| OCD (ZWIK-S) | 44 (66.67) | 44 (30.34) |

*Notes. n* (%) scoring above the respective cut-offs on the symptom severity questionnaires.

Figure S1. The size of each data point (bubble) represents the relative sample size (n) for that specific age group (*N* = 214).
